# Anti-CD20 B Cell Treatment for Relapsing Multiple Sclerosis

**DOI:** 10.3389/fneur.2020.595547

**Published:** 2021-01-22

**Authors:** Charles A. Roach, Anne H. Cross

**Affiliations:** Department of Neurology, Washington University School of Medicine, St. Louis, MO, United States

**Keywords:** multiple sclerosis, anti-CD20 agent, rituximab, ofatumumab, ocrelizumab, ublituximab

## Abstract

Several clinical trials have demonstrated the efficacy of lytic therapies targeting B cells in the treatment of relapsing multiple sclerosis (MS). More modest efficacy has been noted in the primary progressive subtype of MS. Clinical success has increased interest in the role of B cells in the pathogenesis of MS and in ways to potentially improve upon current B cell therapies. In this mini review, we will critically review previous and ongoing clinical trials of anti-CD20 monoclonal antibodies in MS, including rituximab, ocrelizumab, ofatumumab, and ublituximab. Side effects and adverse event profiles will be discussed. Studies examining the proposed mechanisms of action of B cell depleting therapies will also be reviewed.

## Introduction

Four lytic monoclonal antibodies that target the CD20 molecule on B cells have now undergone clinical trials in relapsing multiple sclerosis (RMS). These successful trials showed B cell depletion to be an effective treatment for RMS, focusing scientific attention on the role of B cells in MS. Here we summarize trials using anti-CD20 therapies in RMS and discuss proposed mechanisms of action. Although rituximab (RTX) and ocrelizumab (OCR) have been studied in and OCR is approved for primary progressive MS (PPMS) (1, 2), due to word limitations PPMS studies will not be discussed.

## Clinical Trials of B-Cell Therapies in Relapsing Multiple Sclerosis

### Rituximab

Rituximab is a chimeric mouse-human monoclonal lytic antibody directed at CD20 (3, 4). Two early phase clinical trials of B-cell depletion using RTX as a therapy in relapsing-remitting MS (RRMS) (5, 6) are summarized in Table 1A. An early phase 2 trial in 30 RMS patients with contrast-enhanced lesions (CELs) on brain MRI used RTX at oncology dosing (375 mg/m^2^ weekly, 4 doses) as add-on to injectable disease modifying therapies (DMTs) (5). It showed 88% reduction of number of CELs on brain MRIs after RTX treatment (*p* < 0.0001).

**Table 1A:** A summary of the phase 2 clinic trials of anti-CD20 therapies in RRMS.

**Anti-CD20 mAb**	**Primary endpoint**	**Intervention/control groups**	**Patient number (% Female)**	**Mean age ± Standard deviation**	**ARR [relative reduction] (*p*-value)**	**Disability progression (*p*-value)**	**Mean new CELs (*p*-value)**	**Mean new T2 lesions (*p*-value)**	**References**
RTX (HERMES Trial)	Number of CELs at weeks 12, 16, 20, and 24	RTX 1,000 mg IV	69 (52%)	39.6 ± 8.7	0.20 [50%] (*p* = 0.04)	NR	0.2 (*p* < 0.001)	NS	(6)
		Placebo	35 (29%)	45.5 ± 8.5	0.40	NR	4.5	NS	
RTX	Number of CELs on 3 pre-treatment MRIs vs. 3 post-treatment MRIs	RTX 375 mg/m^2^ weekly × 4 doses as add on to IFNβ or glatiramer acetate.	30 (73.3%)	43.5 (20–50)	0.23	NR	88% reduction post-treatment vs. pre-treatment	NR	(5)
OCR	Number of CELs at weeks 12, 16, 20, 24	OCR 600 mg	55 (64%)	35.6 ± 8.5	0.13 [79%] (*p* = 0.0005)	NR	0.8 (*p* < 0.0001)	0.0 (*p* < 0.0001)	(7)
		OCR 2,000 mg	55 (69%)	38.5 ± 8.7	0.17 [73%], (*p* = 0.0014)	NR	0.8 (*p* < 0.0001)	0.0 (*p* < 0.0001)	
		IFNβ-1a 30 mcg/week IM	54 (59%)	38.1 ± 9.3	0.36 [43%] (*p* = 0.07)	NR	7.2	1.8	
		Placebo infusions days 1 and 15, received OCR at 24 weeks	54 (67%)	38.0 ± 8.8	0.64	NR	6.6	1.4	
OFA	Safety	OFA 100, 300, or 700 mg × 2 doses followed by placebo	26 (61.5%)	36.3 ± 7.9	NS	NS	8–24 weeks 0.04 (*p* < 0.001) 24–48 weeks 0.12	8–24 weeks 0.12 (*p* < 0.001) 24–48 weeks 0.12	(8)
		Placebo followed by OFA 100, 300, or 700 mg × 2 doses	12 (50%)	36.0 ± 9.1	NS	NS	8–24 weeks 9.69 24–48 weeks 0.09	8–24 weeks 10.67 24–48 weeks 0.09	
UTX	B cell depletion	Ublituximab 150 mg IV followed by 400 or 600 mg at weeks 2 and 24	36 (reported in subgroups only)	Reported in subgroups only	0.07	12 weeks 7.0% 24 weeks 17.0%	0.00	24–48 weeks 0.2	(9)
		Placebo	12 (NR)	NR	NR	NR	NR	NR	

*ARR, annualized relapse rate; CEL, contrast enhancing lesions; mAb, monoclonal Antibody; NS, not significant; NR, not reported; OCR, Ocrelizumab; OFA, Ofatumumab; RRMS, Relapsing Remitting MS; UTX, Ublituximab*.

**Table 1B:** A summary of the phase 3 clinical trials of anti-CD20 therapies in RRMS.

**Anti-CD20 mAb**	**Primary endpoint**	**Intervention/Control groups**	**Patient number (% Female)**	**Mean age ± Standard deviation**	**ARR [relative reduction] (*p*-value)**	**Disability progression (*p*-value)**	**Mean new CELs (*p*-value)**	**Mean new T2 lesions (*p*-value)**	**References**
OCR (OPERA I)	ARR	OCR 600 mg every 6 months	410 (65.9%)	37.1 ± 9.3	0.156 [46%] (*p* < 0.0001)	12 weeks pooled for OPERA I and II: 9.1% [60%] (*p* < 0.001)	0.016 (*p* < 0.0001)	0.323 (*p* < 0.0001)	(12)
						24 weeks pooled 6.9% [60%] (*p* = 0.003)			
		IFNβ-1a	411 (66.2%)	36.9 ± 9.3	0.292	12 weeks pooled 13.6%	0.286	1.413	
						24 weeks pooled 10.5%			
OCR (OPERA II)	ARR	OCR 600 mg every 6 months	417 (65%)	37.2 ± 9.1	0.155 [47%] (*p* < 0.0001)	Pooled data above	0.291 (*p* < 0.0001)	0.325 (*p* < 0.0001)	
		IFNβ-1a	418 (67%)	37.4 ± 9.0	0.290	Pooled data above	0.416	1.904	
OFA (ASCLEPIOS I)	ARR	OFA 20 mg every 4 weeks after 20-mg loading doses on days 1, 7, and 14	465 (64.4%)	38.9 ± 8.8	0.11 (*p* < 0.001)	3 months pooled for ASCLEPIOS I and II: 9.3% [66%] (*p* = 0.002)	0.012 (*p* < 0.001)	0.72/year (*p* < 0.001)	(13)
						6 months pooled 7.5% [68%] (*p* = 0.012)			
		Teriflunomide 14 mg daily	462 (68.6%)	37.8 ± 9.0	0.22	3 months pooled 13.7%	0.452	4.0/year	
						6 months pooled 10.6%			
OFA (ASCLEPIOS II)	ARR	OFA 20 mg every 4 weeks after 20-mg loading doses on days 1, 7, and 14	481 (66.3%)	38.0 ± 9.3	0.10 (*p* < 0.001)	Pooled data above	0.032 (*p* < 0.001)	0.64/year (*p* < 0.001)	
		Teriflunomide 14 mg daily	474 (67.3%)	38.2 ± 9.5	0.25	Pooled data above	0.514	4.15/year	

*ARR, annualized relapse rate; CEL, contrast enhancing lesions; mAb, monoclonal Antibody; NS, not significant; NR, not reported; OCR, Ocrelizumab; OFA, Ofatumumab; RRMS, Relapsing Remitting MS; UTX, Ublituximab*.

The HERMES trial was the first double-blind, placebo-controlled trial of RTX in RMS, and demonstrated a significant reduction in CELs (*p* < 0.001) along with an almost 50% reduction in ARR at 48 weeks (*p* = 0.04) vs. placebo (6). CELs remained near zero at 48 weeks, despite no further treatment. These and concurrent observational studies (10) led to increased interest in targeting B cell therapies for the treatment of MS.

As a mouse-human chimeric mAb, development of anti-drug neutralizing mAbs is of concern. RTX is approved for chronic use in several diseases, such as rheumatoid arthritis and Wegener's granulomatosis (but not for MS in the United States). Depending on the disease, the duration of exposure to RTX and the assay methodology, anti-RTX Abs (not all of which were shown to be neutralizing) have been reported in 11 to >50% of people taking RTX chronically (11).

### Ocrelizumab

Ocrelizumab (OCR) is a fully humanized lytic mAb targeting CD20 (14). As a fully humanized mAb, it evokes less anti-drug antibody formation. A phase 2 randomized, double-blind trial compared 600 mg OCR and 2,000 mg OCR delivered intravenously on days 1 and 15 with placebo and with IFNβ-1a 30 micrograms IM weekly 1:1:1:1 in 218 RRMS subjects (Table 1A) (14). The study showed 89% reduction in the total number of CELs at 24 weeks in the OCR 600 mg group (*p* < 0.0001) and 96% reduction in the 2,000 mg group (*p* < 0.0001) vs. placebo. ARR at 24 weeks was 0.13 in the 600 mg dose OCR group, significantly <0.64 for placebo and 0.36 for IFNβ-1a. ARR was also reduced in the 2,000 mg dose OCR group compared to placebo but did not reach statistical significance compared to IFNβ-1a.

Two identically designed phase 3 trials (OPERA I and II) compared OCR with IFNβ-1a in RRMS (12). These multicenter, double-blind, double-dummy, parallel-group trials enrolled 1,656 RRMS patients (OPERA I 821; OPERA II 835) who were randomized 1:1 to receive OCR 600 mg every 24 weeks or IFNβ-1a 44 micrograms subcutaneously injected three times per week over 96 weeks. Patients were between 18 and 55 years of age, with EDSS score ≤ 5.5, and had at least two documented clinical attacks within 2 years, or 1 within 1 year prior to screening. The primary endpoint, ARR, was reduced relative to IFNβ-1a by 46 and 47%, CELs were reduced by 94 and 95%, the number of new and/or enlarging T2 lesions was reduced by 77 and 83%, and rate of brain volume loss was reduce by 22.8 and 14.9% in OPERA I and OPERA II, respectively. In pre-specified pooled analyses, the percentage of subjects with 3 months confirmed disability worsening (CDW) and 6-months CDW was 40% lower in the OCR groups compared with IFNβ-1a (Table 1B).

### Ofatumumab

Ofatumumab (OFA) is fully human mAb targeting CD20-expressing cells (15). An early small phase 2, placebo-controlled, double-blind trial demonstrated no safety concerns in 38 RRMS subjects OFA or placebo 2 weeks apart (Table 1A) (8). This study was followed by a larger, phase 2, multicenter, double-blind study of OFA (17) that enrolled 232 RRMS subjects randomized to receive subcutaneous OFA 3, 30, or 60 mg every 12 weeks, OFA 60 mg every 4 weeks, or placebo every 12 weeks or every 4 weeks for 24 weeks. After the 12-weeks placebo-controlled period, the placebo group received a single 3 mg OFA dose while the remaining subjects continued their original dose of OFA. At 12 weeks, mean cumulative new CELs was reduced 65% for all OFA groups compared with placebo (*p* < 0.001). *Post-hoc* analysis excluding weeks 1–4 estimated a ≥90% reduction in CELs at 12 weeks for all groups that received ≥30 mg OFA.

Two identically designed multicenter, double-blind, double-dummy, parallel-group phase 3 trials compared 20 mg subcutaneous OFA every 4 weeks to 14 mg oral teriflunomide daily, randomized 1:1 (13). The trials enrolled 927 and 955 subjects, respectively. Subjects enrolled were mostly RRMS, but a small percentage (5.9% in ASCLEPIOS I and 5.6% in ASCLEPIOS II) had active secondary progressive MS (SPMS). Inclusion required at least 2 documented clinical attacks within 2 years or 1 within 1 year of screening, or a CEL on MRI in the year before randomization. EDSS scores at baseline were ≤ 5.5. Both studies met their primary endpoint with similar and significant relative reductions in ARR in the OFA arms. ARR for the OFA arms were reduced by over 50% relative to the teriflunomide arms (0.11 vs. 0.22 and 0.10 vs. 0.25 in ASCLEPIOS I and II, respectively). Significant (>30%) reductions in CDW relative to teriflunomide were seen in pooled 3 and 6-months CDW analyses (10.9 vs. 15.0% and 8.1 vs. 12.0%, respectively). Six-months sustained disability improvement favored the OFA arm in both trials but did not reach statistical significance. Significant reductions in mean CELs per scan (0.01 vs. 0.45, and 0.03 vs. 0.51 in ASCLEPIOS I and II, respectively) and new or enlarging T2 lesions per year (0.72 vs. 4.00, and 0.64 vs. 4.15, respectively) were seen in the OFA vs. teriflunomide groups. Serum neurofilament light chain concentrations were reduced in the OFA arm relative to teriflunomide arm by 7% at month 3, 27% at month 12, and 23% at month 24 in ASCLEPIOS I and by 11% at month 3, 26% at month 12, and 24% at month 24 in ASCLEPIOS II (Table 1B).

### Ublituximab

Ublituximab (UTX) is a chimeric mAb targeting CD20 that has been glycoengineered to remove sugar molecules, resulting in enhanced lytic potency (18) (Table 2). In a phase 2, placebo-controlled trial of UTX, 48 RRMS subjects were randomized 3:1 to receive UTX IV or placebo on day 1, day 15, and week 24 (9). No CELs were seen at weeks 24 and 48, and a 10.6% reduction in T2 lesion volume was seen in the UTX group vs. placebo. Further studies of this drug are ongoing. As a chimeric mAb, the potential development of anti-UTX Abs will need to be monitored.

**Table 2:** A summary of anti-CD20 antibody type, target, and mechanisms of lysing B lymphocytes.

**mAb**	**Antibody type and target**	**Mechanism of action**	**References**
RTX	Mouse/human chimeric IgG1 mAb that targets CD20	Lyses B cells by direct signaling of apoptosis, complement activation, and ADCC	(3, 4)
OCR	Humanized IgG1 mAb that targets CD20	Lyses B cells by ADCC and complement mediated lysis	(14)
OFA	Fully human IgG1 mAb that targets CD20	Lyses B cells by CDC and ADCC	(15)
UTX	Mouse/human chimeric IgG1 mAb glycoengineered for high affinity for FcγRIIIa	Enhanced lyses of B cells via ADCC compared to RTX Similar effects on apoptosis and CDC compared to RTX	(16)

*Ab, Antibody; ADCC, antibody dependent cellular cytotoxicity; CDC, complement dependent cytotoxicity; mAb, monoclonal antibody; OCR, Ocrelizumab; OFA, Ofatumumab; RTX, Rituximab; UTX, Ublituximab*.

## Safety Considerations

### Infusion Reactions

Infusion reactions were the most common adverse events in the OCR and RTX phase 3 trials. These were mostly mild to moderate in severity and decreased in rate and severity with subsequent dosing. There were no fatal or life-threatening reactions in the phase 3 trials. Longer-term safety event reporting data suggest that infusion related reactions occur at similar rates in MS patients treated with RTX and OCR at 4.82 and 4.76%, respectively (19). Injection reactions with subcutaneous OFA occurred at 16.1 and 24.1% in the ASCLEPIOS I and II trials, respectively and were largely confined to the first dose.

### Infections Including Progressive Multifocal Leukoencephalopathy (PML) and COVID-19

The most common minor infections seen in the phase 3 trials of RTX, OCR, and OFA were upper respiratory infections, nasopharyngitis, and urinary tract infections (UTIs). These occurred at similar rates in the anti-CD20 groups of these trials. Recent real-world safety reporting data showed nearly a 2-fold higher rate of minor infections in OCR compared to RTX with significantly higher rates of UTIs, nasopharyngitis, and oral herpes (19).

As of end of January 2020, nine cases of definite PML according to AAN criteria have been reported in MS patients receiving OCR (20). Seven of these cases were in anti-JCV antibody positive patients who had previously received natalizumab; one case occurred in a patient previously treated with fingolimod. One reported case of PML occurred in a patient treated with OCR that had not received prior DMTs, but this was confounded by older patient age (78 years) and low absolute lymphocyte counts prior to OCR treatment.

A potential risk of anti-CD20 therapies in people with MS infected with SARS-CoV-2 has been reported. A retrospective study of 784 MS patients with SARS-CoV-2 infection conducted by the Italian MS and COVID-19 registry found increased risk of severe COVID-19 in people treated with OCR or RTX with an odds ratio of 2.59 (*p* = 0.002) (21). A multi-center retrospective French study with only 347 total patients did not find an association of severe COVID-19 with anti-CD20 therapies (22). The North American COViMS Registry has reported 858 MS patients with SARS-CoV-2 infection (23). Multivariable logistic regression analysis demonstrated an OR of 2.31 (*p* < 0.002) for those on anti-CD20 to have higher chance of death, ICU or hospitalization compared with those on other DMTs. None of these early reports have true denominators. Research in this area is ongoing; COViMS Registry and several other worldwide efforts continue to accrue data.

### Malignancy

Fifteen malignancies were observed over the 96-weeks study periods in patients randomized to OCR compared to 4 in the IFNβ-1a or placebo groups in the phase 3 trials in RRMS and PPMS. The latest OCR package insert (dated May 2020) states that “an increased risk of malignancy, including breast cancer, may exist with OCREVUS.” We recommend that all patients taking anti-CD20 mAbs closely adhere to standard cancer screening guidelines, including periodic skin checks for skin cancers.

## Mechanism of Action of Anti-CD20 Monoclonal Antibodies in Multiple Sclerosis

The most consistent laboratory abnormality found in MS patients is increased intrathecal production of antibodies (Abs), which is most sensitively detected as cerebrospinal fluid (CSF)-restricted oligoclonal bands (OCB). CSF-restricted OCBs are present in more than 90% of persons with definite MS (24). Elevated levels of CSF IgG and IgM, and number of OCBs have been correlated with worse MS prognosis (25, 26). This indirectly implicates B lymphocytes, as B cells produce Abs. However, plasma cells (which differentiate from B cells but do not express CD20) are the long-lived cells that produce most Abs.

Some insights into the mechanisms of action of B cell depletion with anti-CD20 mAb derived from the Phase 2 study we performed using oncologic doses of RTX (375 mg/m^2^ weekly × 4 weeks). In this MRI-blinded open-label study (5), 26 of the 30 subjects underwent CSF and blood collection before and 6 months after RTX treatment. B cells declined in the CSF after RTX treatment in 20 of the 26 subjects (*p* < 0.0001 by Wilcoxon matched pairs test). In the remaining six subjects, B cells were undetectable in CSF prior to or after RTX. CSF Abs as measured by IgG index, IgG concentration, and oligoclonal band number did not decline 6 months post-RTX (27). Because the major producers of Ab are plasma cells that do not express CD20, this was not surprising. Given the rapidity of the beneficial effects of anti-CD20 mAbs in RRMS, these studies suggested that reduced Ab levels are unlikely to be critical for the mechanism of action of anti-CD20 mAb therapies.

**Figure 1:**
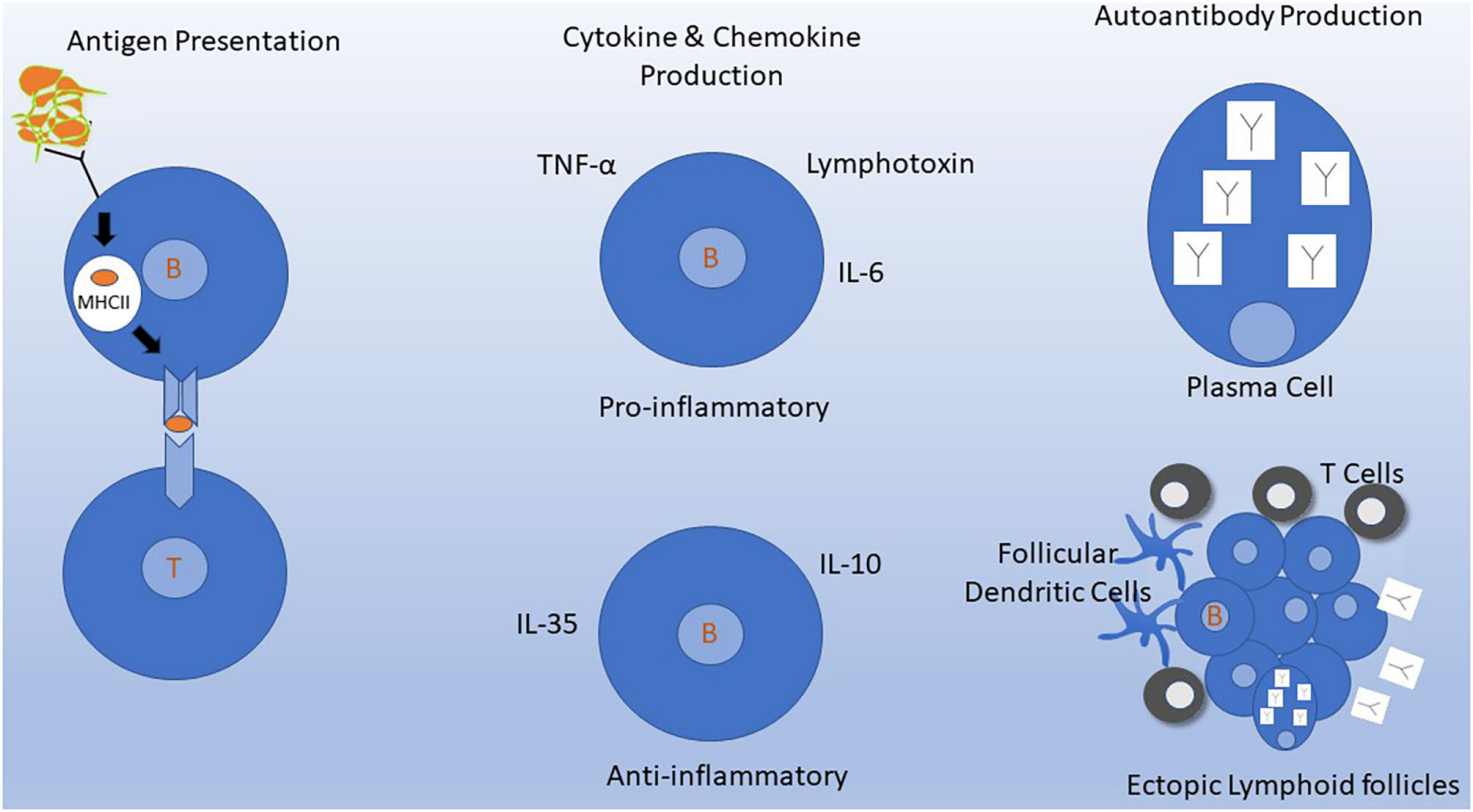
Mechanisms of B cells in MS pathogenesis. Depletion of B cells by targeting CD20 molecules on the B cell surface inhibits several of these mechanisms either directly or indirectly. (Left) Antigen Presentation. B cells efficiently process and present antigens to T cells. B cells constitutively express MHC molecules and T cell costimulatory molecules that allow for interaction and activation of autoreactive T cells. This process occurs in lymph nodes and may also occur in meningeal ectopic lymphoid follicles in the meninges (bottom right). (Middle) Naive and memory B cells can produce cytokines and chemokines that have various downstream effects on the immune system. B cell signaling via B cell receptor engagement and CD40 leads to production of several pro-inflammatory cytokines and down regulation of IL-10. (Upper Right) Intrathecal antibody production detected by CSF oligoclonal banding is the most consistent laboratory abnormality identified in MS. Antibodies are produced primarily by plasma cells which do not express CD20 and thus are not depleted by anti-CD20 monoclonal antibodies in the short term. (Lower Right) Ectopic lymphoid follicles containing B cells, T cells, follicular dendritic cells, and plasma cells develop at sites of chronic inflammation. In MS, these can develop in the meninges, where their presence has been associated with a worse clinical course.

However, B cells have other functions aside from their role in Ab production (Figure 1). B cells comprise several subtypes, such as naïve and memory B cells, and including B cells that produce proinflammatory (e.g., IL-6), or anti-inflammatory (e.g., IL-10) cytokines (28). Memory B cells are strongly implicated in the underlying pathophysiology of MS (29, 30). For this reason, studies to test the possibility of tailoring anti-CD20 treatments to target continued absence of circulating memory B cells are being pursued (31). Bar-Or and colleagues showed that B cell signaling via the combination of B cell receptor engagement and CD40 leads to production of several pro-inflammatory cytokines (e.g., lymphotoxin and tumor necrosis alpha), while reducing B cell production of IL-10 (32). Two chemokines, CXCL13 and CCL19, were significantly decreased (*P* = 0.002, *P* = 0.03, respectively) in post-RTX CSF (27). Lysis of B cells using anti-CD20 eliminates their production of cytokines and chemokines and may contribute to the mechanism of action of anti-CD20 treatments.

T cells were also reduced in the CSF of 81% of subjects 6 months after RTX treatment (27, 33, 34). The mean reduction of CSF B cells was 95% and of CSF T cells was 55%. T cells were reduced in the CSF to a larger degree than the 12% reduction observed in blood, suggesting reduced T cell trafficking into the CNS. Activated T cells express CXCR5, the receptor for the chemoattractant factor CXCL13. CXCL13 is increased in CSF during several CNS inflammatory conditions, including MS (35) and CXCL13 was reduced 6 months post-RTX (27). Reduced trafficking of T cells into the CSF appears to be an indirect consequence of B cell elimination, especially since B cells themselves do not produce CXCL13 (36). A better understanding of the manifold effects of anti-CD20 mAb therapy in MS is expected from a multicenter longitudinal study that is underway (NCT02688985).

B cells are constitutively able to process and present antigen to T cells, and they are extraordinarily efficient at this when presenting their own cognate antigen to T cells recognizing the same antigen (37, 38). B cells that target myelin recognize it via surface B cell receptors, which enables efficient antigen capture of a self-antigen that is at low concentration. As B cells constitutively express MHC-I and MHC-II and the T cell costimulatory molecules CD86 and CD80, they are ready to process and present antigens to pathogenic autoreactive T cells (39). A process by which B cells that capture low concentration myelin antigens and then serve as antigen-presenting cells (APCs) to activate myelin-reactive T cells is postulated to be a trigger of MS activity. The interaction of T with B cells further cross-activates the B cells. Several groups have reported evidence that this process can occur in deep cervical lymph nodes (40, 41). In established MS, the process may occur in meningeal ectopic lymphoid-like structures (42).

Yet another mechanism through which anti-CD20 mAbs may act is the elimination of CD20-expressing T cells. A small proportion of circulating T cells express surface CD20; these cells are eliminated by anti-CD20 treatments. CD20^+^ T cells comprise only 3–5% of circulating T cells of healthy persons (43), but comprise a slightly higher proportion (up to 10%) in MS patients (44, 45). CD4^+^ and CD8^+^ CD20^+^ T cells produce pro-inflammatory cytokines, such as interferon gamma, TNF-alpha and GM-CSF, which could contribute to MS pathogenesis (45). In MS, CSF T cells are enriched for those expressing CD20^+^, but are still <50% of CSF T cells (45) suggesting that the CSF T cell reduction observed after RTX treatment cannot be fully explained by their lysis by anti-CD20 mAb.

An early report using the lytic anti-CD19 mAb, inebilizumab, in MS showed benefit on MRI activity to a similar strong degree as seen with anti-CD20 therapies (46). This study may provide insights, as the CD19 molecule is expressed on B cells but not on T cells. Results using inebilizumab hinted that lysis of CD20^+^ T cells is not responsible for all beneficial effects of anti-CD20 mAb treatments.

## Other Emerging B Cell Therapies in MS

Success in therapeutics with targeting CD20 on B cells has raised interest in other mechanisms to target B cells, but the results have not always been as expected. Early on, the drug atacicept was tried in two studies, one in optic neuritis patients in hope of preventing MS development and the second in MS patients in a phase 2 trial. Atacicept is a human recombinant fusion protein that comprises the binding portion of a receptor for both B-Lymphocyte Stimulator (B-LyS) and A PRoliferation-Inducing Ligand (APRIL), two important factors supporting B cell maturation and survival. Unexpectedly atacicept led to more attacks (47). These ill-fated trials may in fact point to the importance of memory B cells in MS activity because, while inhibiting late state B cells and plasma cells, atacicept selectively spares memory B cells (48).

Bruton's tyrosine kinase (BTK) is a cytoplasmic enzyme important for B cell signaling; inhibition of BTK results in B cell inhibition (49). A phase 2 placebo-controlled trial in RMS patients of varying doses of the oral BTK inhibitor evobrutinib showed fewer CELS at the higher doses compared to placebo (50). Currently, this and several other BTK inhibitors are being studied in MS patients. Early reports suggest moderate efficacy of BTK inhibitors that is not as profound as that seen with anti-CD20 mAb therapies.

## Conclusions

In summary, eliminating circulating CD20^+^ B cells leads to a profound reduction in MS clinical and MRI activity in RMS patients. B cells likely contribute to MS pathogenesis in several ways, including their enhancement of T cell activation and proliferation. B cells are critical for capturing and presenting low concentration antigens, such as myelin proteins to T cells. B cells also likely contribute to MS pathogenesis by direct and indirect production of pro-inflammatory cytokines and chemokines. Elimination of pro-inflammatory CD20^+^ T cells may also play a role. The mechanism by which B cells contribute to MS activity appears to be independent of their role in Ab production. Collecting longer-term safety data will be important to determine the safety of using these therapies chronically. Studies to determine exactly how B cell depletion inhibits MS activity will undoubtedly lead to better understanding of MS pathogenesis.

## Author Contributions

CR analyzed the clinical trials data and safety information and drafted the manuscript on these topics. AC analyzed the mechanisms of action data and drafted the manuscript, revised the draft of clinical trials and safety data, and approved the final draft. All authors contributed to the article and approved the submitted version.

## Conflict of Interest

AC has received research grants from EMD Serono and Genentech/Roche, and fees for serving on scientific advisory boards for Biogen, Celgene, EMD Serono, Genentech, Novartis, and Roche, Janssen Pharmaceuticals and Greenwich Biosciences. The remaining author declares that the research was conducted in the absence of any commercial or financial relationships that could be construed as a potential conflict of interest.
